# Protective role of the CD73-A2AR axis in cirrhotic cardiomyopathy through negative feedback regulation of the NF-κB pathway

**DOI:** 10.3389/fimmu.2024.1428551

**Published:** 2024-07-17

**Authors:** Ning Zhao, Zhenhao Shao, Guoqing Xia, Huanhuan Liu, Lei Zhang, Xiaoxi Zhao, Shipeng Dang, Lingling Qian, Wentao Xu, Zhiming Yu, Ruxing Wang

**Affiliations:** ^1^ Wuxi School of Medicine, Jiangnan University, Wuxi, China; ^2^ Inflammation and Immune Mediated Diseases Laboratory of Anhui Province, Anhui Institute of Innovative Drugs, School of Pharmacy, Anhui Medical University, Hefei, China; ^3^ Wuxi Medical Center of Nanjing Medical University, Wuxi, China

**Keywords:** cirrhotic cardiomyopathy, inflammation, apoptosis, CD73, A2AR, NF-κB pathway

## Abstract

**Background:**

Myocardial inflammation and apoptosis induced by cirrhosis are among the primary mechanisms of cirrhotic cardiomyopathy. CD73, a common extracellular nucleotidase also known as 5’-nucleotidase, is associated with the progression of inflammation and immunity in multiple organs. However, the mechanism by which CD73 contributes to myocardial inflammation and apoptosis in cirrhosis remains unclear.

**Methods:**

In this study, a cirrhotic cardiomyopathy model in mice was established by bile duct ligation. Myocardial-specific overexpression of CD73 was achieved by tail vein injection of AAV9 (adeno-associated virus)-cTNT-NT5E-mCherry, and cardiac function in mice was assessed using echocardiography. Myocardial inflammation infiltration and apoptosis were evaluated through pathological observation and ELISA assays. The expression of CD73, A2AR, apoptotic markers, and proteins related to the NF-κB pathway in myocardial tissue were measured.

**Results:**

In the myocardial tissue of the cirrhotic cardiomyopathy mouse model, the expression of CD73 and A2AR increased. Overexpression of CD73 in the myocardium via AAV9 injection and stimulation of A2AR with CGS 21680 inhibited myocardial inflammation and cardiomyocyte apoptosis induced by cirrhosis. Additionally, overexpression of CD73 suppressed the activation of the NF-κB pathway by upregulating the expression of the adenosine receptor A2A.

**Conclusion:**

Our study reveals that the CD73/A2AR signaling axis mitigates myocardial inflammation and apoptosis induced by cirrhosis through negative feedback regulation of the NF-κB pathway.

## 1 Introduction

Cirrhotic cardiomyopathy (CCM) refers to the chronic heart dysfunction that occurs in patients with cirrhosis who have no prior history of cardiovascular diseases (1). The standard therapy for end-stage liver disease is liver transplantation, and cardiovascular function is a major determinant of prognosis in cirrhotic patients undergoing transplantation (2). Recent studies have shown that CCM is associated with increased mortality and morbidity following major interventions such as liver transplantation and trans jugular intrahepatic portosystemic shunt (TIPS). Concurrently, CCM occurs in up to 50% of patients with cirrhosis (3). Therefore, the development of drugs to improve cardiovascular dysfunction related to the advanced liver disease is urgently needed.

It has been established that immune infiltration and activation of the inflammatory process in myocardial tissue are key pathogenic mechanisms of CCM (4). Research by Lee et al. have demonstrated that an increase in monocytes/macrophages in the hearts of cirrhotic rats, along with reduced myocardial contractile function (5). The recruitment of monocytes/macrophages can be significantly improved by blocking their mobilization with gadolinium chloride, leading to enhanced cardiac systolic and diastolic functions. Additionally, the increase in nuclear factor-κB (NF-κB) and pro-inflammatory cytokines such as tumor necrosis factor-α (TNFα), interleukin (IL)-1β, and IL-6 is closely related to heart function (6, 7).

Danger signals such as adenosine triphosphate (ATP) are released when cells undergo death and damage, and extracellularly, they act as immune stimulatory signals that promote inflammatory responses (8). The Ectonucleotidases CD39/nucleoside triphosphate diphosphohydrolase-1 and CD73/ecto-5’-nucleotidase on the cell surface can break down extracellular ATP into adenosine (9). Adenosine is a key endogenous molecule that participates in various physiological and pathological processes by binding to and activating G protein-coupled adenosine receptors (ARs). There are four subtypes of adenosine receptors: A1AR, A2AR, A2BAR, and A3AR, with A1AR and A2AR having a higher affinity for adenosine (10). Studies have shown that an increase in CD73-positive lymphocytes in patients with cardiac arrest is associated with better survival rates, possibly through its anti-inflammatory effects (11). Meanwhile, T cell-expressed CD73 can modulate the inflammatory response after myocardial infarction through A2AR, reducing the release of pro-inflammatory cytokines (12). Furthermore, CD73 also plays a role in monocytes and mesenchymal stem cells (MSCs), promoting an anti-inflammatory state in the heart after myocardial infarction (13). However, research has shown that upregulation of CD73 in epicardial cells (EPDCs) may lead to pro-inflammatory and pro-fibrotic responses in the heart (14). This indicates that CD73 has a complex role in the regulation of inflammation and tissue repair after myocardial injury, depending on its expression in different cell types and environmental conditions.

CCM is a type of metabolic cardiomyopathy (15). A plethora of current research studies have underscored the intimate association between CD73 and cardiac diseases. Particularly, in instances of myocardial injury, there is a tendency for CD73 to increase compensatory. This phenomenon has been observed and documented across various pathological states of the heart. Such compensatory upregulation may signify a self-preservation mechanism of the heart in response to injury, aiming to mitigate myocardial damage by promoting anti-inflammatory and tissue repair processes. However, the precise mechanisms of action of CD73 in cardiac diseases and its potential therapeutic value warrant further in-depth investigation to elucidate. Although the function of CD73 has been validated in various myocardial injury models, its role and mechanism in metabolic cardiomyopathies have not been reported to date. After conducting bioinformatics analysis, we observed significant enrichment of cardiac genes in the apoptotic and purinergic signaling pathways three days post-biliary ligation. Concurrently, there was a notable alteration in the expression of the CD73 gene (**Supplementary Figure S1A**). Considering the significant role of CD73 in inflammation-related diseases and cardiovascular diseases, We hypothesize that CD73 serves as a negative feedback regulator in the pathogenesis of CCM. We believe that overexpression of CD73 may help alleviate cardiac inflammation and apoptosis caused by liver cirrhosis, and may further improve the diastolic dysfunction of the heart induced by liver cirrhosis. This study aims to explore the role and molecular mechanisms of CD73 in CCM.

## 2 Materials and methods

### 2.1 Animals

Male C57BL/6 J mice (6-8 weeks old, 20-24 g) were obtained from Changzhou Cavens Laboratory Animal Co. Ltd. and were housed in a suitable environment for three days before the start of the experiment. All animal experiments were conducted in accordance with the regulations of the Animal Ethics Committee of Jiangnan University and the guidelines for the care and use of laboratory animals. The bile duct ligation (BDL) procedure was performed as previously described (16), with a sham surgery group (exposure of the abdomen without bile duct ligation) serving as the control group, and subsequent experiments were conducted 14 days post-surgery. Following bile duct ligation (BDL) surgery, CGS 21680, a specific agonist of the adenosine A2A receptor (A2AR), was administered intraperitoneally at a dosage of 1 mg/kg for a continuous period of 14 days.

### 2.2 Echocardiography

Mice were anesthetized with isoflurane inhalation and positioned supine on the surgical table. The anterior chest skin was cleaned and an appropriate amount of transmission gel was applied. Echocardiographic examinations were performed using a Vevo 2100 high-resolution small animal ultrasound system with an FMS-250 transducer, with the transducer depth set between 2.0 and 2.5 cm and a frequency range of 13 to 24 MHz. M-mode ultrasound images were used to observe the heart structure. The following parameters were derived of the left ventricle from Teichholz calculation: cardiac output, ejection fraction, fractional shortening, mitral valve early (E) to late (A) ventricular filling velocities (E/A ratio).

### 2.3 ALT/AST activity assay

ALT/AST kits were purchased from Nanjing Jiancheng Bioengineering Research Institute. Serum separated from whole blood was centrifuged at 3000 rpm for 30 minutes. The levels of alanine aminotransferase (ALT), aspartate aminotransferase (AST), total cholesterol (TC), and total triglycerides (TG) in the serum were measured using the ALT/AST/TG/T-CHO kit. The analysis of serological indicators was strictly conducted according to the instructions, and the optical density values at 510 nm were determined using a Thermomax microplate reader (Bio-Tekel, USA).

### 2.4 ELISAs

Frozen heart tissues were initially processed in cold PBS. After homogenization and centrifugation (4°C, 3000 rpm, 10 minutes), the levels of TNF-α, IL-6, IL-1β, and BNP in the mouse myocardial tissue were strictly determined according to the requirements of the ELISA kit (Wuhan Ailab Science and Technology Co., Ltd.). The optical density was then measured using a microplate reader as needed. The kits used were as follows: E-el-m0037c with a coating antibody of rat monoclonal antibody, detection antibody of goat polyclonal antibody, and antigen of recombinant mouse IL-1β; E-EL-M0044C with a coating antibody of rat monoclonal antibody, detection antibody of goat polyclonal antibody, and antigen of recombinant mouse IL-6; E-el-m0049c with a coating antibody of rat monoclonal antibody, detection antibody of goat polyclonal antibody, and antigen of recombinant mouse TNF-α.

### 2.5 Immunohistochemistry

Immunohistochemistry was conducted on paraffin sections to detect the expression of CD45 and CD73 using a microwave-assisted antigen retrieval method. The sections were incubated overnight at 4°C with rabbit anti-CD45 (dilution 1:50) and rabbit anti-CD73 (dilution 1:300, CST, D7F9A). Following incubation with the secondary antibody and 3,3’-diaminobenzidine tetrahydrochloride, the slides were counterstained with hematoxylin. The stained tissues were observed under an inverted fluorescence microscope (OLYMPUS IX83, Tokyo, Japan) or scanned with Panoramic MIDI (3D HISTECH, Hungary) to digitize the images for further analysis. The entire slide was scanned and viewed using CaseViewer slicing software.

### 2.6 Histopathology

Samples were first fixed in 10% formalin for a period of 24 to 48 hours and then embedded in paraffin for the purpose of histopathological examination. Thin slices (5 μm) were subjected to hematoxylin and eosin (H&E) staining for histopathological evaluation. The stained specimens were digitized using a Panoramic MIDI scanner (3D HISTECH, Hungary) and analyzed with CaseViewer slicing software.

### 2.7 Western blot analysis

Heart tissue proteins were extracted using RIPA buffer (Beyotime, Shanghai), followed by quantification with a NanoDrop 2000 (Thermo, California, USA). After ensuring the protein extracts met quantitative criteria, proteins were separated via SDS-PAGE electrophoresis and nonspecific binding sites were blocked by incubating in 5% skimmed milk at room temperature for 3 hours. The blocked PVDF membrane was incubated with primary antibodies at 4°C in a refrigerator overnight and subsequently with secondary antibodies (goat anti-rabbit or anti-mouse, Bios, Beijing) for 24 hours. Imaging was performed using an ECL luminescence kit (Thermo Scientific, USA), with ImageJ software employed for results analysis. This procedure was conducted three times independently. Moreover, the Bax/Bcl-2 ratio was utilized to denote changes in apoptotic proteins. The primary antibodies included: GAPDH (1:1000, Proteintech, Wuhan, 60004-1-Ig), CD73 (1:1000, CST, D7F9A), A2AR (1:000, CST,94871S), p-P65 (1:800, CST, 3033S), P65 (1:800, CST, 8242T), Iκb-α (1:1000, CST, 4812S), p-Iκb-α (1:10000, CST, 2859S), Cleaved-Caspase3 (1:1000, Abcam, ab214430), Bax (1:800, Abcam, ab53154), and Bcl-2 (1:1000, Abcam, ab194583).

### 2.8 Real-time PCR analysis

RNA was isolated from heart tissue utilizing Trizol reagent (Invitrogen, USA). Subsequently, the isolated RNA was reverse transcribed into cDNA employing the TAKARA kit (Qiagen, Japan). The mRNA levels of CD73, TNF-α, IL-6, and IL-1β were assessed utilizing a specific program setup (96-well format). The relative expression levels of mRNA were determined using the 2-ΔΔCT approach. The primer sequences employed in this study are listed in **Table 1**.

**Table 1:** Primer information.

Gene	Forward	Reverse
**CD73**	**CTGAGCGCTCTACTACCACA**	**AACAGCACGTTGGGTTCTTC**
**A2AR**	**GGAGCCAGAGCAAGAGGTAT**	**ACGACTCTGCATCTCCCAAA**
**IL-1β**	**GCAACTGTTCCTGAACTCAACT**	**ATCTTTTGGGGTCCGTCAACT**
**IL-6**	**TAGTCCTTCCTACCCCAATTTCC**	**TTGGTCCTTAGCCACTCCTTC**
**TNF-α**	**CCCTCACACTCAGATCATCTTCT**	**GCTACGACGTGGGCTACAG**

### 2.9 Adeno-associated virus injection

The purified adeno-associated virus vector encoding NT5E was provided by Universal Biotechnology (Shanghai, China). AAV9 (adeno-associated virus)-cTnT-NT5E-mCherry was administered at a concentration of 1 × 10^10 pfu/mL via a single tail vein injection to enhance the expression of NT5E, with AAV9-cTnT-mCherry serving as a negative control. According to the manufacturer’s instructions, each mouse was given 100 µL as an effective infective dose, and the infection efficiency was verified in subsequent analyses.

### 2.10 Statistical analysis

Experimental results are shown as the mean ± standard error of the mean (SEM) using GraphPad Prism 7.0. In our study, when the data from two groups are normally distributed and exhibit homogeneity of variances, we employ the t-test. For three or more groups that satisfy the condition of homogeneity of variances, we utilize Analysis of Variance (ANOVA). This particular experiment solely encompasses the use of the independent samples t-test and one-way ANOVA. Notably, a P-value of less than 0.05 is considered to denote a statistically significant difference.

## 3 Results

### 3.1 Liver characterization of mice 14 days after bile duct ligation

**Figure 1:**
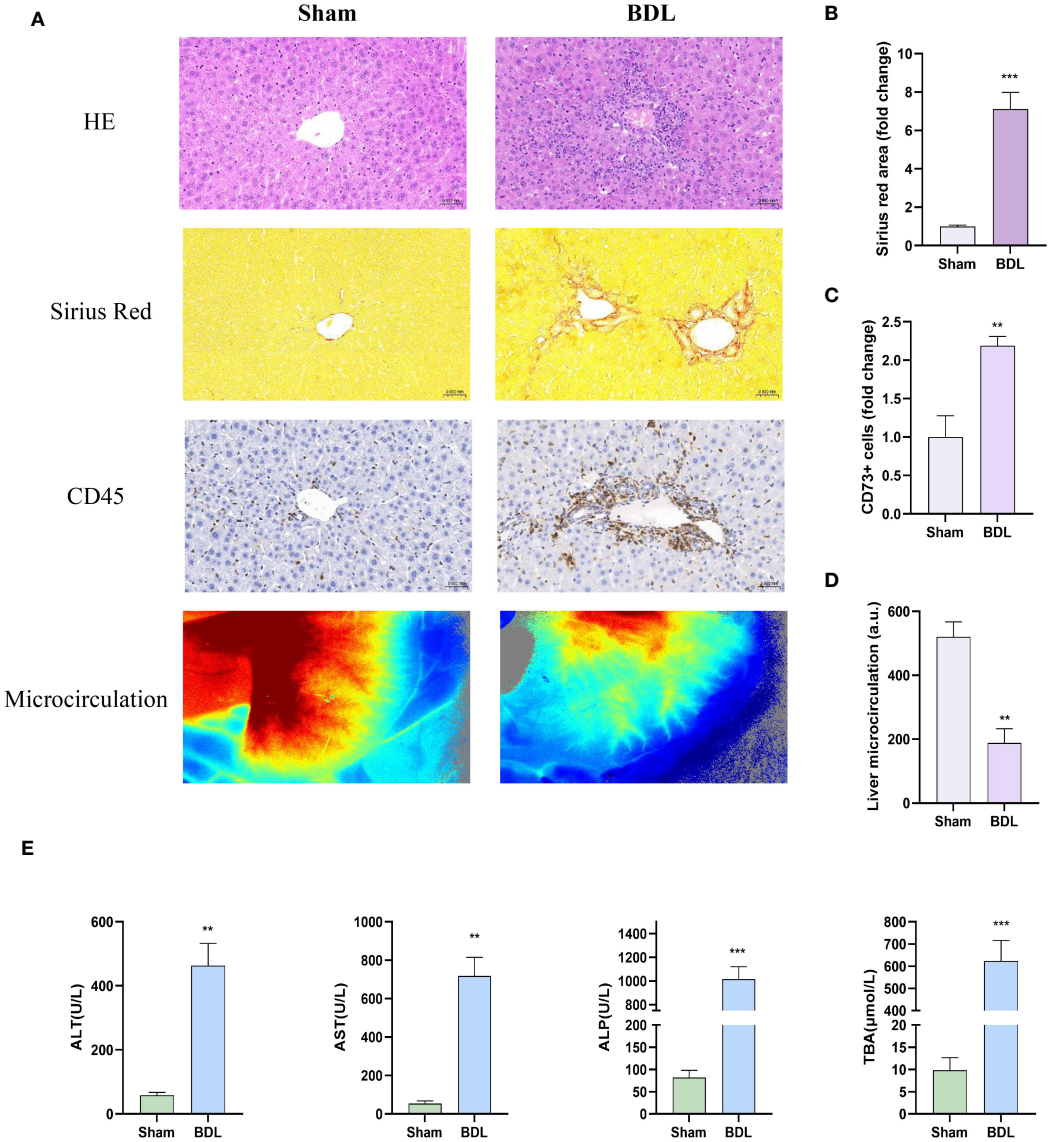
Characterization of livers in mice 14 days after bile duct ligation **(A)** Representative images of hematoxylin-eosin histology (400x), Sirius red staining (Magnification: 400x), CD45 immunohistochemistry (400x) and liver microcirculation. **(B)** Quantification results of Sirius red staining (n=6/group). **(C)** Quantification results of CD45+ cell (n=4-6/group). **(D)** Analysis of liver microcirculation. (n=4-6/group). **(E)** Levels of serum ALT, AST, ALP and TBA (n=4-6/group). BDL: bile duct ligation **p<0.01, ***p<0.001 vs Sham.

Pathological liver evaluation showed that tissue disintegration, leukocyte infiltration, and fibrosis in the BDL-treated group were significantly greater than in the Sham group (**Figure 1A**). Furthermore, we performed quantitative analyses of CD45 immunohistochemical staining, Sirius Red staining, and the average perfusion of liver blood flow (**Figures 1B–D**). Additionally, serum analysis revealed that the levels of ALT, AST, ALP and TBA in the BDL group mice were significantly higher than those in the sham surgery group (**Figure 1E**), indicating a marked decrease in liver function due to BDL surgery.

### 3.2 Heart characterization of mice 14 days after bile duct ligation

**Figure 2:**
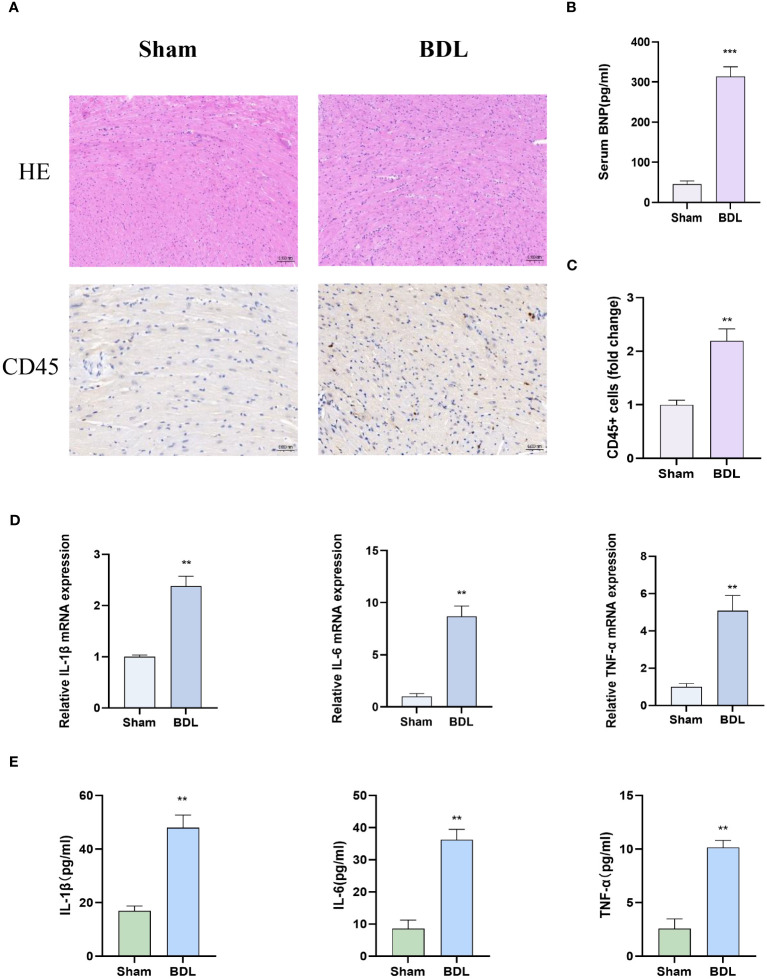
Heart characterization of mice 14 days after bile duct ligation **(A)** Representative images of hematoxylin-eosin histology (400x) and CD45 immunohistochemistry (Magnification: 400x). **(B)** Level of serum BNP (n=4-6/group). **(C)** Quantification results of CD45+ cell (n=4-6/group). **(D)** Relative mRNA expression of IL-1β, il-6 and TNF-α(n=4-6/group). **(E)** Levels of myocardium IL-1β, il-6 and TNF-α (n=4-6/group). **p<0.01, ***p<0.001 vs Sham.

We then assessed cardiac pathology, and HE staining results showed that compared to the sham group, the BDL group had disordered myocardial cell arrangement and increased leukocyte accumulation indicated by CD45 staining (**Figure 2A**). The Sirius Red staining results indicate that biliary ligation for two weeks did not induce myocardial fibrosis (**Supplementary Figure S1B**). Immunohistochemistry and Immunofluorescence results showed that, compared to the sham group, mice that underwent bile duct ligation exhibited a significant increase in myocardial apoptosis (**Figure 2C**). Concurrently, Q-PCR and ELISA detected a significant increase in pro-inflammatory cytokine levels in myocardial tissue and serum BNP levels in the BDL group, suggesting that BDL surgery led to increased myocardial tissue inflammation and impaired cardiac function (**Figures 2B, D, E**).

### 3.3 CD73 was more highly expressed in mice after bile duct ligation

**Figure 3:**
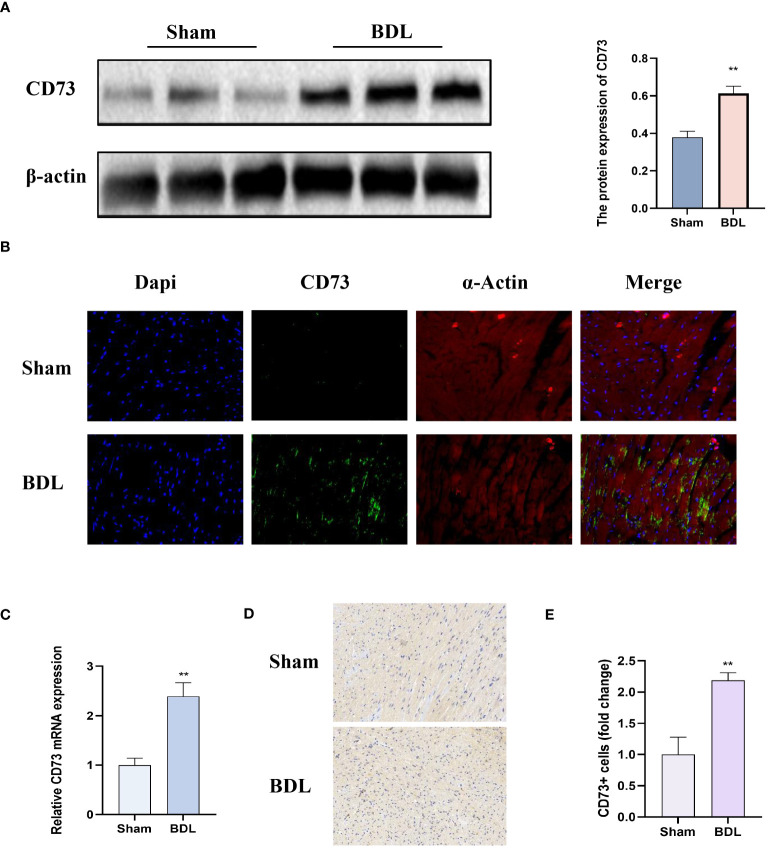
CD73 expression was significantly higher in mice after bile duct ligation **(A)** The protein expression of CD73 in heart tissue(n=4/group). **(B)** Representative double immunofluorescence images of CD73 (green) (400x) and α-actin (red) (400x) in Sham and BDL group are presented(n=4/group). **(C)** Relative mRNA expression of CD73 in heart tissue(n=4/group). **(D)** Representative images of IHC of CD73 (200x) in Sham and BDL group are presented. **(E)** Quantification results of CD73+ cell (n=4/group). **p < 0.01 vs Sham.

We first detected the protein expression CD73 in myocardial tissue. The results indicate that compared to the sham group, mice that underwent bile duct ligation showed a significant increase in CD73 expression in myocardial tissue (**Figure 3A**). Immunohistochemistry and Immunofluorescence analysis also showed that compared to the sham group, mice that underwent BDL surgery had significantly increased expression of CD73 in myocardial tissue (**Figures 3B, D, E**). Moreover, the mRNA expression of CD73 in myocardial tissue also increased (**Figure 3C**). These results indicate that the CD73 is upregulated in the animal model of cirrhotic cardiomyopathy, suggesting that CD73 may be associated with myocardial inflammation and apoptosis induced by cirrhosis.

### 3.4 Overexpression of CD73 improves diastolic dysfunction caused by liver cirrhosis by inhibiting the NF-kB pathway through upregulation of A2AR expression

**Figure 4:**
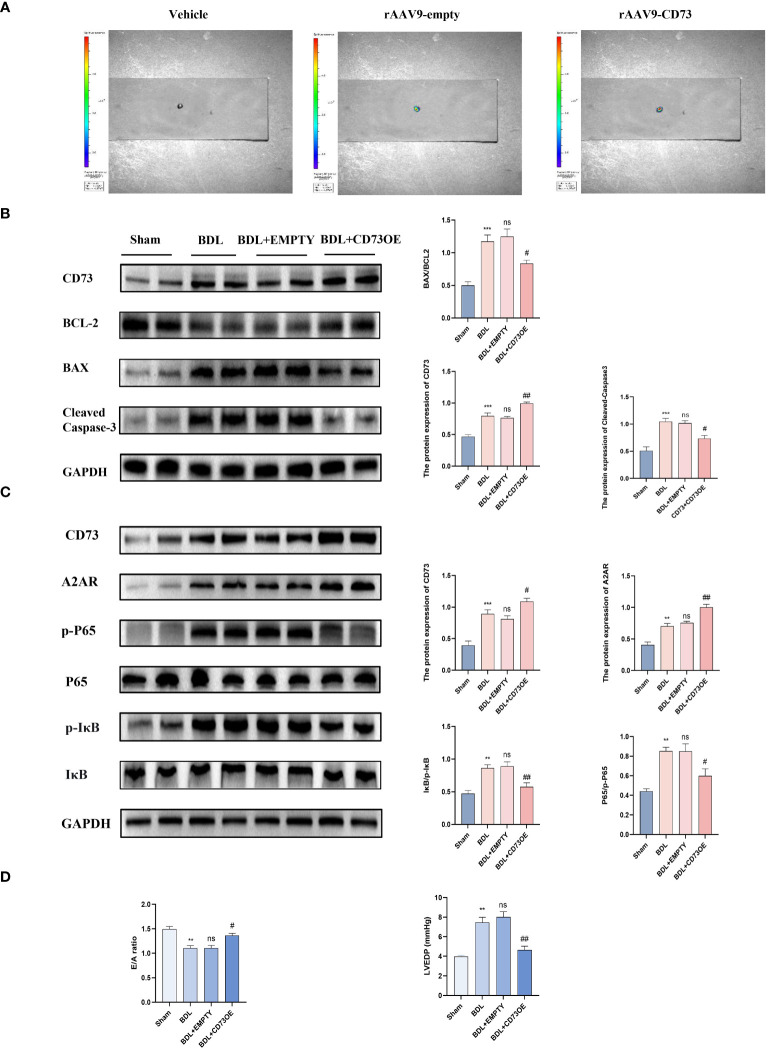
Overexpression of CD73 improves diastolic dysfunction caused by liver cirrhosis by inhibiting the NF-kB pathway and by upregulation of A2AR expression **(A)** Representative high-efficiency transduction of in heart tissue. **(B)** Protein expression of CD73, cleaved caspase-3 and the ratio of Bax/Bcl-2 in heart tissues(n=4/group). **(C)** Protein expression of CD73, A2AR, IκB, p- IκB, P65 and p-P65 (n=4/group). **(D)** Diastolic functional indices: E/A ratio on echocardiography and LV end-diastolic pressure (LVEDP) (n=4/group). **p<0.01, ***p<0.001 vs Sham, #p<0.05, ##p<0.01 vs BDL+EMPTY. ns indicates P > 0.05, suggesting no significant difference between the indicated groups.

To verify the role of CD73 in myocardial inflammation and apoptosis caused by liver fibrosis, mice were injected with AAV9 (adeno-associated virus)-NT5E-cTNT-mCherry to specifically increase the expression of CD73 in myocardial tissue, with the empty AAV9-cTNT-mCherry vector serving as the adeno-associated virus control. The successful infection of mouse hearts by the adeno-associated virus was determined by observing the enhanced fluorescence protein (mCherry) in fresh hearts using small animal *in vivo* imaging (**Figure 4A**). Protein immunoblot analysis indicated that compared to the model group receiving the empty vector, BDL group mice with AAV9-Ctnt-NT5E-mCherry had increased expression of CD73 and A2AR, and a significant reduction in the ratio of phosphorylated IκB to IκB (p-IκB/IκB) and phosphorylated P65 to P65 (p-p65/P65), along with reduced expression of the apoptosis-related protein cleaved Caspase3 and a decreased Bax/Bcl-2 ratio (**Figures 4B, C**). Additionally, results from small animal echocardiography also demonstrated that overexpression of CD73 improved diastolic dysfunction caused by liver cirrhosis (**Figure 4D**).

### 3.5 Overexpression of CD73 ameliorates cardiac inflammation and cardiomyocyte apoptosis due to liver cirrhosis

**Figure 5:**
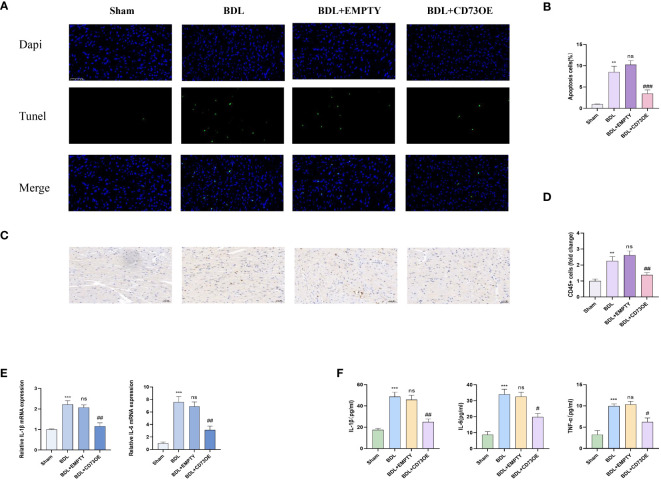
Overexpression of CD73 reduces cardiac inflammation and cardiomyocyte apoptosis caused by liver cirrhosis **(A)** Representative immunofluorescence images of TUNEL (green) (400x) in Sham, BDL, BDL+EMPTY and BDL+CD73OE group are presented. **(B)** Quantification results of TUNEL+ cell(n=4/group). **(C)** Representative images of CD45 immunohistochemistry(400x) **(D)** Quantification results of CD45+ cell(n=4/group). **(E)** Relative mRNA expression of IL-1β, il-6 and TNF-α in Sham, BDL, BDL+EMPTY and BDL+CD73OE group(n=4/group). **(F)** Levels of myocardium IL-1β, il-6 and TNF-αin Sham, BDL, BDL+EMPTY and BDL+CD73OE group(n=4/group). **p<0.01, ***p<0.001 vs Sham, #p<0.05, ##p<0.01, ###p<0.001 vs BDL+EMPTY. ns indicates P > 0.05, suggesting no significant difference between the indicated groups.

Inflammation and apoptosis are key factors leading to heart dysfunction in cirrhotic cardiomyopathy (16). Fourteen days after the mice underwent bile duct ligation surgery, the levels of myocardial injury, inflammation, and apoptosis in each group were detected. Histologically, BDL group mice injected with AAV9-cTNT-NT5E-mCherry showed reduced leukocyte infiltration and alleviated myocardial apoptosis compared to those injected with the empty vector (**Figures 5A–D**). Additionally, compared to mice injected with the empty vector, those injected with AAV9-cTNT-NT5E-mCherry had significantly lower transcriptional levels of pro-inflammatory cytokines in myocardial tissue (**Figure 5E**). The results of the ELISA experiments were similar (**Figure 5F**). These results indicate that cardiac-specific overexpression of CD73 can alleviate myocardial inflammation and apoptosis caused by liver cirrhosis.

### 3.6 Activating A2AR meliorates cardiac inflammation and cardiomyocyte apoptosis due to liver cirrhosis

**Figure 6:**
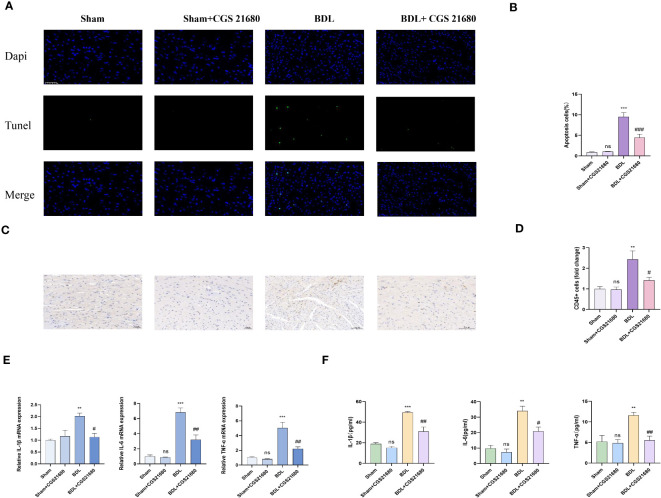
Activating A2AR meliorates cardiac inflammation and cardiomyocyte apoptosis caused by liver cirrhosis **(A)** Representative immunofluorescence images of TUNEL(green) (400x) in Sham, Sham+CGS 21680, BDL and BDL+ CGS 21680 group are presented. **(B)** Quantification results of TUNEL+ cell(n=4/group). **(C)** Representative images of CD45 immunohistochemistry **(D)** Quantification results of CD45+ cell (400x) (n=4/group). **(E)** Relative mRNA expression of IL-1β, il-6 and TNF-αin Sham, Sham+CGS 21680, BDL and BDL+ CGS 21680 group(n=4/group). **(F)** Levels of myocardium IL-1β, il-6 and TNF-αin Sham, Sham+CGS 21680, BDL and BDL+ CGS 21680 group(n=4/group). **p<0.01, ***p<0.001 vs Sham, #p<0.05, ##p<0.01, ###p<0.001 vs BDL. ns indicates P > 0.05, suggesting no significant difference between the indicated groups.

CD73 catalyzes the extracellular hydrolysis of ATP to generate adenosine, which in turn can activate four distinct adenosine receptors. Western blot analysis has shown that upon overexpression of CD73, the expression levels of adenosine receptors A1, A2A, and A3 are all upregulated to varying degrees, while the expression of A2B receptor remains unchanged (**Supplementary Figure S1C**). Among these, the change in A2AR receptor expression is the most pronounced. Consequently, we have identified A2AR as the primary downstream effector of CD73. Fourteen days after bile duct ligation surgery, the levels of myocardial inflammation and apoptosis in each group were detected. Histologically, compared to the BDL group, the BDL + CGS 21680 group showed reduced leukocyte infiltration and alleviated myocardial apoptosis (**Figures 6A–D**). Additionally, compared to the BDL group, the BDL + CGS 21680 group had significantly lower mRNA levels of pro-inflammatory cytokines in myocardial tissue (**Figure 6E**). The results of the ELISA experiments were consistent (**Figure 6F**). Overall, these results reveal that activation of the adenosine receptor A2A mitigates the myocardial inflammation and apoptosis response caused by cirrhosis.

### 3.7 Activating A2AR improves diastolic dysfunction caused by liver cirrhosis by inhibiting the NF-kB pathway

**Figure 7:**
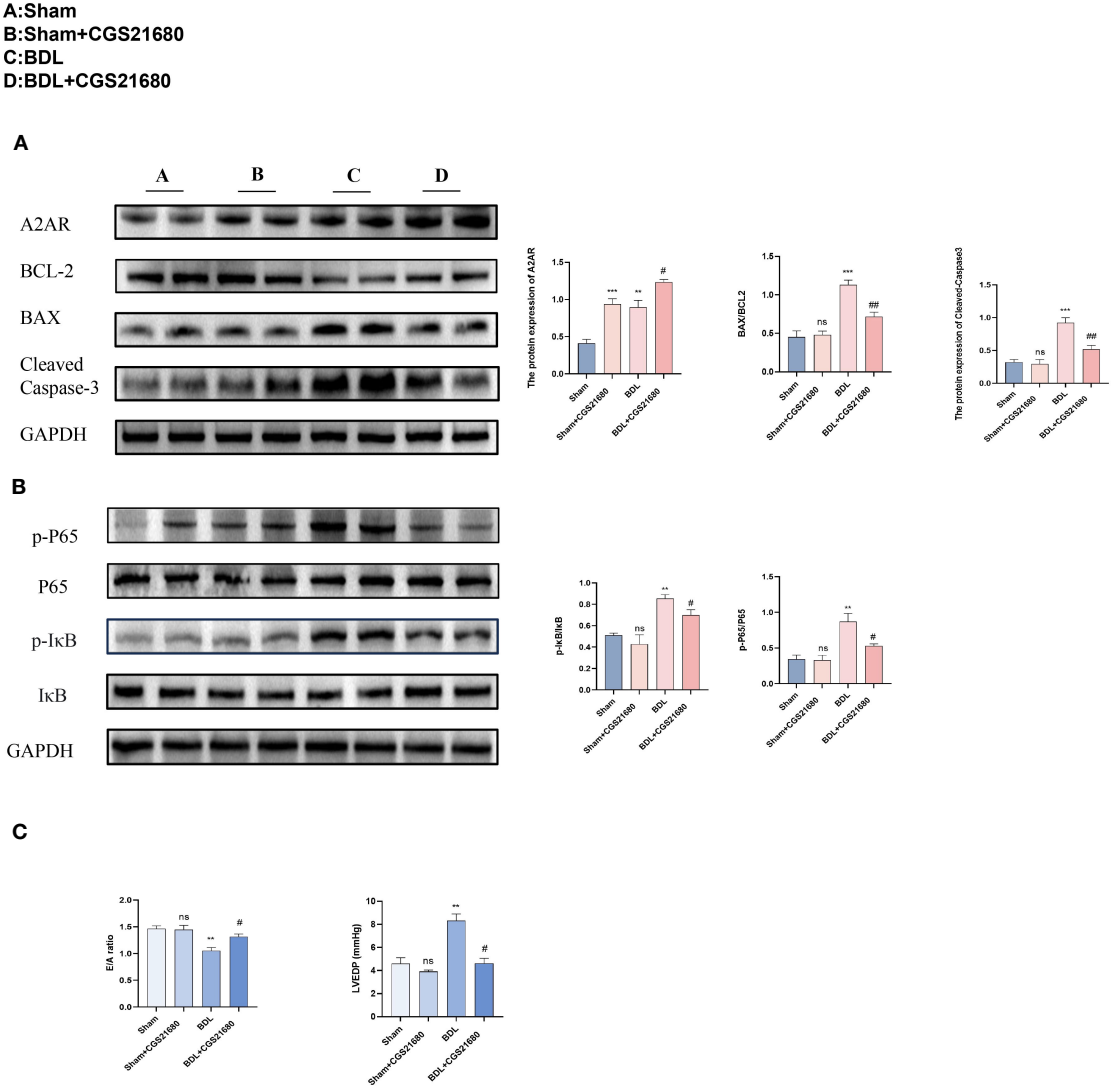
Activating A2AR improves diastolic dysfunction caused by liver cirrhosis by inhibiting the NF-kB pathway **(A)** Protein expression of A2AR, cleaved caspase-3 and the ratio of Bax/Bcl-2 in heart tissue(n=4/group). **(B)** Protein expression of IκB, p- IκB, P65 and p-P65 (n=4/group). in heart tissue(n=4/group). **(C)** Diastolic functional indices: E/A ratio on echocardiography and LV end-diastolic pressure (LVEDP) (n=4/group). **p<0.01, ***p<0.001 vs Sham, #p<0.05, ##p<0.01 vs BDL+EMPTY. ns indicates P > 0.05, suggesting no significant difference between the indicated groups.

To investigate the biological function of A2AR in CCM, mice were administered CGS 21680 (a specific agonist of A2AR, 1mg/kg) via intraperitoneal injection to increase the expression of A2AR in the heart (17). Protein immunoblot analysis indicated that compared to the BDL group, the BDL + CGS 21680 group had increased expression of A2AR, and reduced expression of the apoptosis-related protein cleaved Caspase3 and a decreased Bax/Bcl-2 ratio, along with a significant reduction in the ratio of phosphorylated IκB to IκB (p-IκB/IκB) and phosphorylated P65 to P65 (p-p65/P65) (**Figures 7A, B**). Mouse heart function was again assessed through echocardiography. Results from small animal echocardiography also demonstrated that overexpression of CD73 improved diastolic dysfunction caused by liver cirrhosis (**Figure 7C**).

## 4 Discussion

The pathological progression of chronic liver disease is a complex physiological process involving the liver response to various pathological stimuli, including chronic alcoholic liver disease, non-alcoholic fatty liver disease, infectious agents, and autoimmune processes (18). These pathological stimuli ultimately lead to the development of liver fibrosis and cirrhosis. For patients with end-stage liver failure, liver transplantation is currently the only recognized effective treatment method (19). However, the success of liver transplantation depends not only on the transplant surgery itself but also on the patients’ long-term health status postoperatively. Current research indicates that the progression of cirrhotic cardiomyopathy has been an important determinant of patient survival after liver transplantation (15). Cirrhotic cardiomyopathy is a distinct heart disease characterized by covert impairments in cardiac contractile and diastolic functions, accompanied by a hyperdynamic circulation state, and a sluggish response of the heart to various stress stimuli and electrophysiological abnormalities. This condition may affect up to 50% of patients with cirrhosis (20).

Although there are currently various drugs for the clinical treatment of chronic heart failure, there are no special methods developed for cirrhotic cardiomyopathy, a particular liver complication. At the same time, there is a lack of research on the pathological mechanisms of cirrhotic cardiomyopathy (21). This indicates the need for further research on cirrhotic cardiomyopathy to develop new therapeutic targets for cardiac and vascular complications caused by liver failure (22).

Bile duct ligation is one of the common animal models for liver fibrosis. Recent studies have demonstrated that bile duct ligation for two weeks in mice is a reliable model for cirrhotic cardiomyopathy (4). Our experimental results also showed that after two weeks of bile duct ligation, significant inflammation and fibrosis were found in mice liver. Concurrently, echocardiography and serum BNP results indicate that the mice have heart dysfunction alongside liver fibrosis.

The purinergic signaling pathway is closely related to inflammation (23). CD73 functions by hydrolyzing ATP extracellularly to produce adenosine, which plays a pivotal role in various diseases (24). Our results show that in the animal model of cirrhotic cardiomyopathy, the changes in CD73 and A2AR in myocardial tissue are the most significant, likely due to A2AR’s high affinity for adenosine. The source of CD73 in the heart is still under debate; although Zhao et al. (25) suggest that T cells are the main source of CD73, there is also substantial evidence indicating that various cells from myocardial tissue express CD73. Given the unclear role of CD73 in CCM, our team first studied its physiological role in CCM by myocardial-specific overexpression of CD73, and the source issue will also be a focus in our further study, which will provide a basis for precise treatment with CD73 as a therapeutic target.

Previous studies have confirmed that inflammation and apoptosis play a key role in the progression of cirrhotic cardiomyopathy. Cytokines, especially tumor necrosis factor (TNF-alpha), are one of the pathogenic factors of cirrhotic cardiomyopathy (26, 27). We found that myocardial-specific overexpression of CD73 significantly improved leukocyte infiltration in myocardial tissue caused by cirrhosis, reduced the production of cardiac inflammatory cytokines, and myocardial cell apoptosis, thereby improving the heart function of mice. In addition, we also found that the expression of A2AR increased after overexpressing CD73. Combined with the simultaneous changes of CD73 and A2AR in the animal model and multiple literature reports, we speculate that A2AR is the main downstream receptor of CD73.

After activating A2AR in animals, we also observed the improvement of serum biochemical indicators related to cirrhotic cardiomyopathy. Nuclear factor-κB (NF-κB), as a key factor in cytokine regulation, leads to impaired heart function in various heart disease models. Liu et al. (28) confirmed that NF-κB contributes to the development and progression of cirrhotic cardiomyopathy. Several recent studies have reported that cAMP can negatively feedback regulate the NF-κB signaling pathway. A2AR is a G protein-coupled receptor, and many studies have confirmed that the activation of A2AR can significantly increase cAMP levels (29, 30). There are also investigations indicating that A2AR is closely related to the NF-κB signaling pathway (31, 32). Our study also shows that activating A2AR can restore myocardial tissue cAMP levels and inhibit the activation of the NF-κB signaling pathway. At the same time, overexpressing CD73 can also inhibit the activation of the NF-κB signaling pathway.

Cirrhotic cardiomyopathy (CCM), a recently redefined comorbidity of liver cirrhosis, exhibits a pronounced increase in incidence particularly during the advanced stages of cirrhosis and the transplant period. The medical community has progressively deepened its understanding of CCM, recognizing its profound threat to patient health; however, to date, no specific therapeutic agents have been developed to target CCM.

Our experimental studies have elucidated the anti-inflammatory and anti-apoptotic effects of the CD73-A2AR axis in animal models, which significantly ameliorate the cardiac dysfunction induced by cirrhosis. This discovery presents an auspicious perspective: the CD73-A2AR axis may emerge as a novel therapeutic target for the treatment of CCM.

Prior to the clinical translation of these research findings, it is imperative to initially validate the functional efficacy of the CD73-A2AR axis in human subjects. Given the intricate roles of CD73-A2AR in various organs and pathologies, further investigation into its specific mechanisms in CCM is warranted. Additionally, to enhance therapeutic efficacy and mitigate potential side effects, it is essential to refine the drug delivery strategies based on CD73, ensuring precise targeting of the cardiac tissue.

We harbor great anticipation for the future development in the field of CCM treatment. Through relentless exploration and innovation, we are confident in our ability to offer more efficacious and safer therapeutic options for CCM patients, thereby improving their quality of life and ushering in groundbreaking advancements in this domain.

## 5 Conclusion

In this study, we successfully established a mouse animal model for CCM and for the first time, explored the functional role and potential mechanism of the CD73-A2AR axis in cirrhosis-triggered myocardial inflammatory reactions and cellular apoptosis *in vivo*. We found that BDL resulted in higher expression of CD73 and A2AR in mouse myocardial tissue. In addition, CD73 mainly inhibits inflammatory reactions by activating the A2AR receptor, which participates in the negative feedback regulation of the inflammatory response. In summary, CD73-mediated inhibition of inflammatory reactions significantly suppresses apoptosis in myocardial cells. Although there are still some issues to be resolved, CD73 may serve as a potential therapeutic target for cirrhotic cardiomyopathy.

## Data availability statement

The original contributions presented in the study are included in the article/**Supplementary Material**. Further inquiries can be directed to the corresponding author.

## Ethics statement

All animal experiments were conducted in accordance with the regulations of the Animal Ethics Committeeof Jiangnan University and the guidelines for the care and use of laboratory animals (registration number JN. No 20211215m0901001).

## Author contributions

ZN: Writing – original draft. ZS: Writing – original draft. GX: Writing – original draft. HL: Writing – original draft. LZ: Writing – original draft. XZ: Writing – original draft. SD: Writing – original draft. LQ: Writing – original draft. WX: Writing – original draft. ZY: Writing – review & editing. RW: Writing – review & editing.

## Supplementary material

Supplementary Figure 1 Enrichment Analysis of Cardiac Gene Expression and Sirius Red Staining Results of Cardiac Tissue after two weeks of biliary ligation **(A)** Enrichment Analysis of Cardiac Gene Expression. **(B)** Representative images of Sirius red staining (Magnification: 400x). **(C)** Protein expression of CD73, A1AR, A2AR, A2BAR and A3AR in heart tissue(n=6/group). **p < 0.01 vs Sham.
