# Supplementary figures and images for: Protective role of the CD73-A2AR axis in cirrhotic cardiomyopathy through negative feedback regulation of the NF-κB pathway
Source: Front Immunol. 2024 Jul 17;15:1428551. doi: 10.3389/fimmu.2024.1428551 (PMC11288852; doi:10.3389/fimmu.2024.1428551)

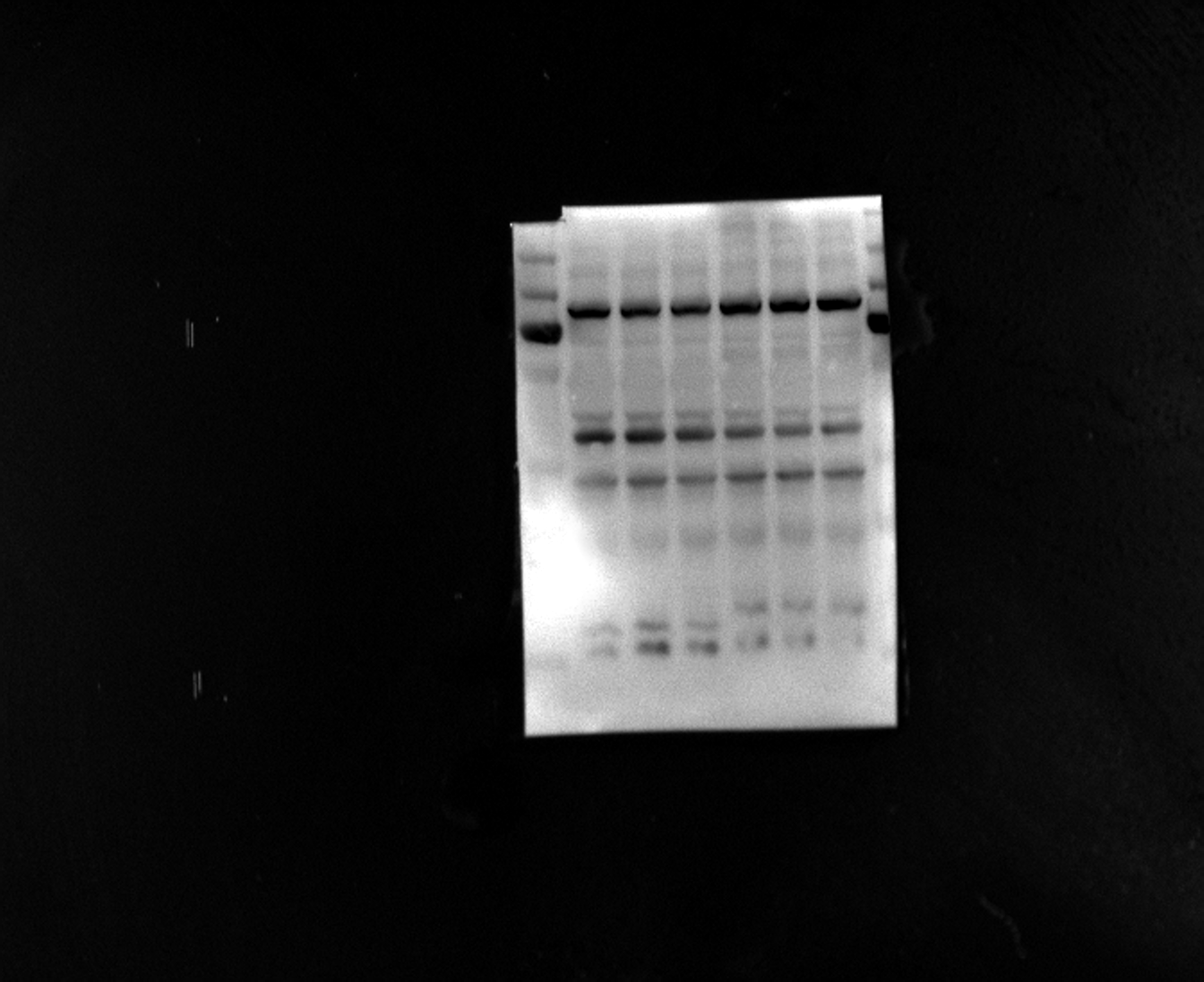

Supplement: Supplementary file 1 [file DataSheet_1.zip › A1AR.Tif]

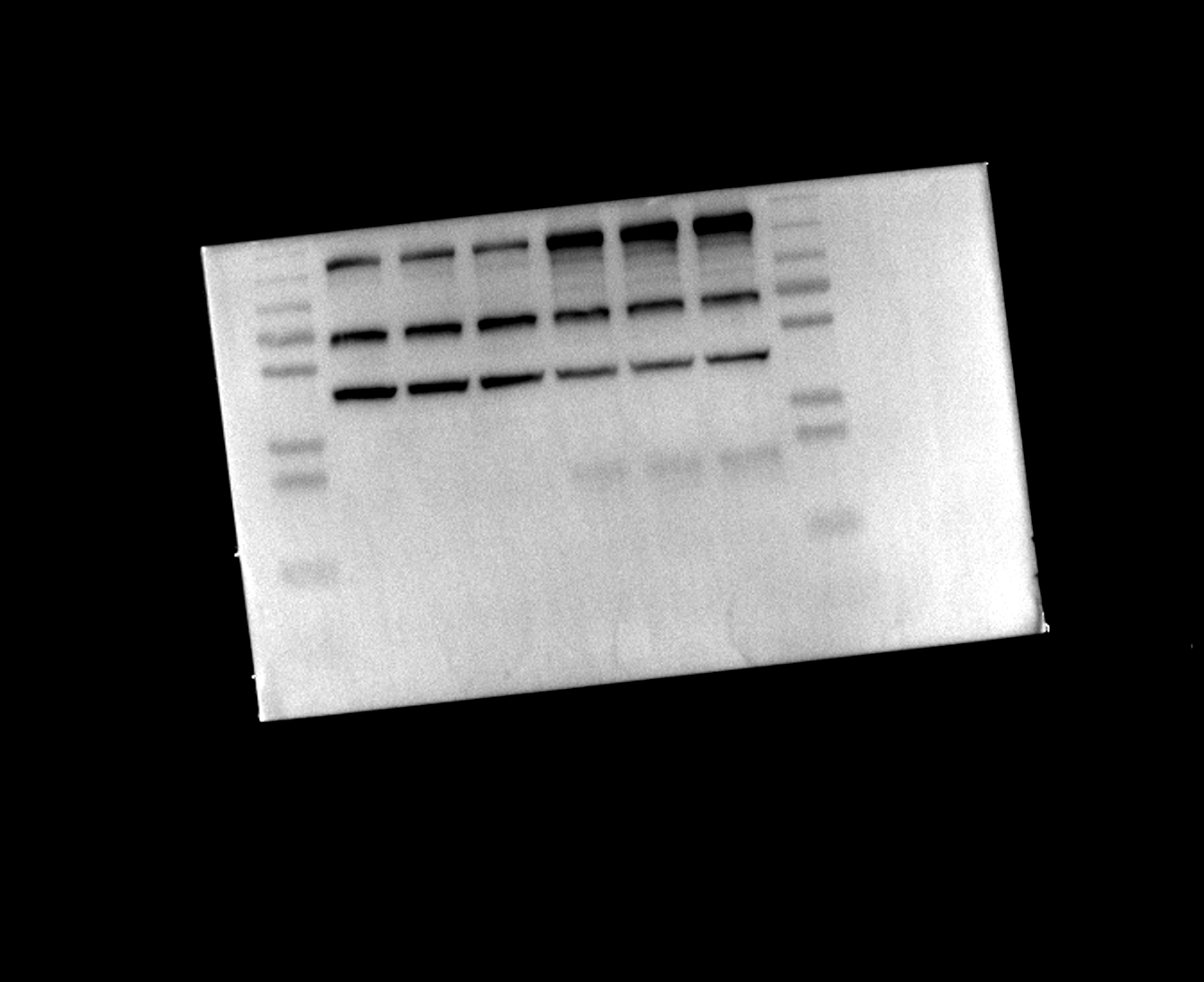

Supplement: Supplementary file 1 [file DataSheet_1.zip › A2AR.Tif]

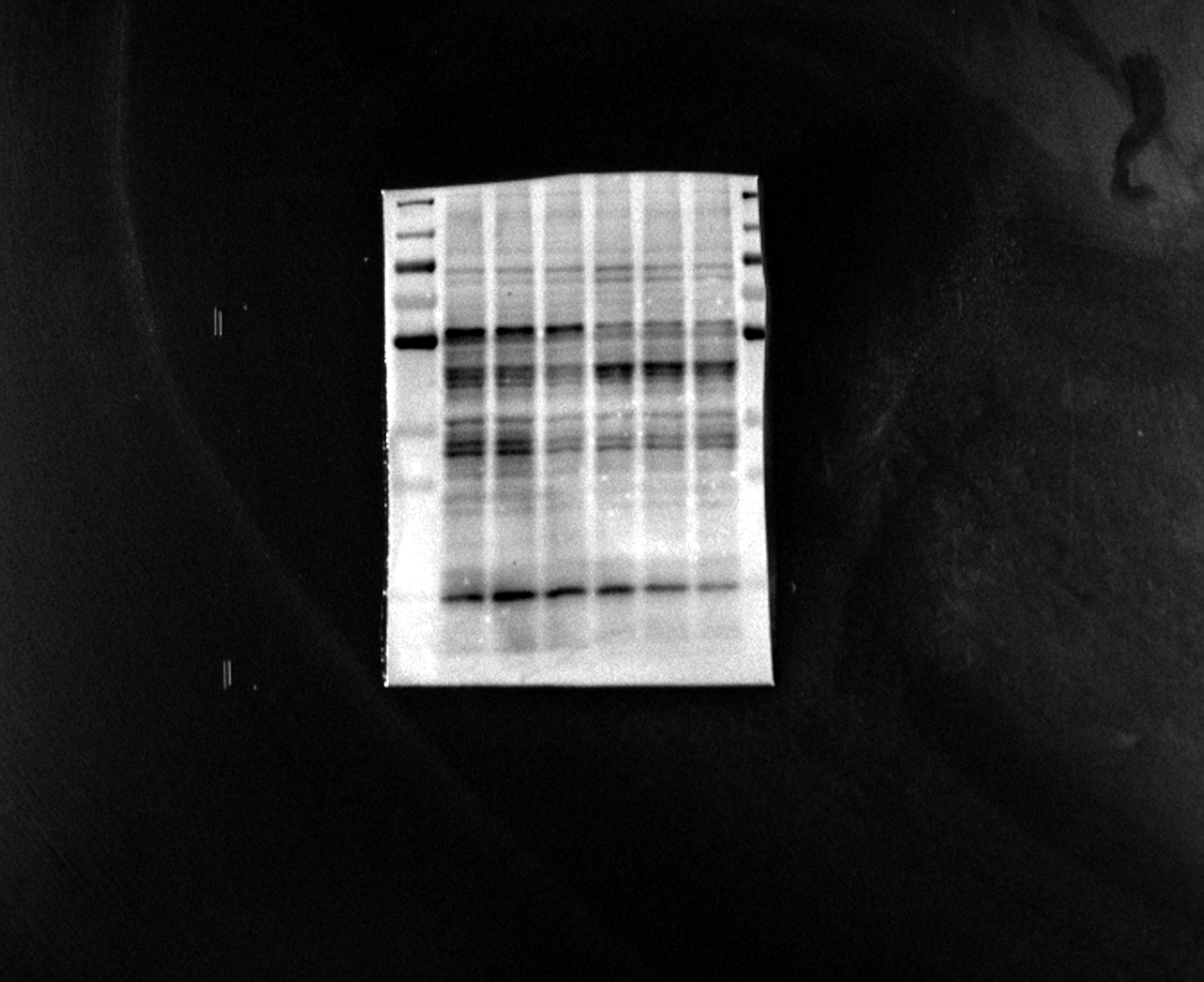

Supplement: Supplementary file 1 [file DataSheet_1.zip › A2BAR.Tif]

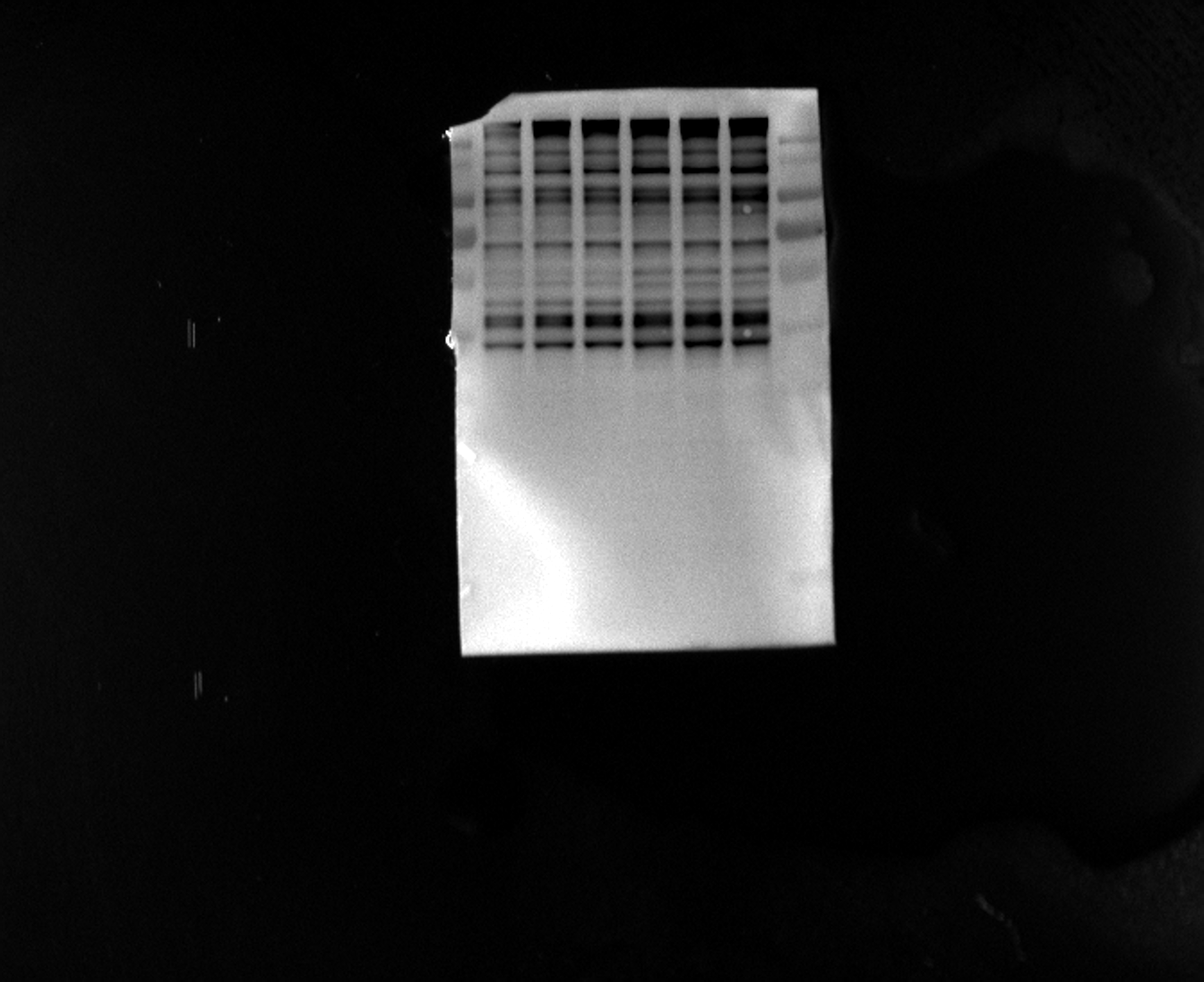

Supplement: Supplementary file 1 [file DataSheet_1.zip › A3AR.Tif]

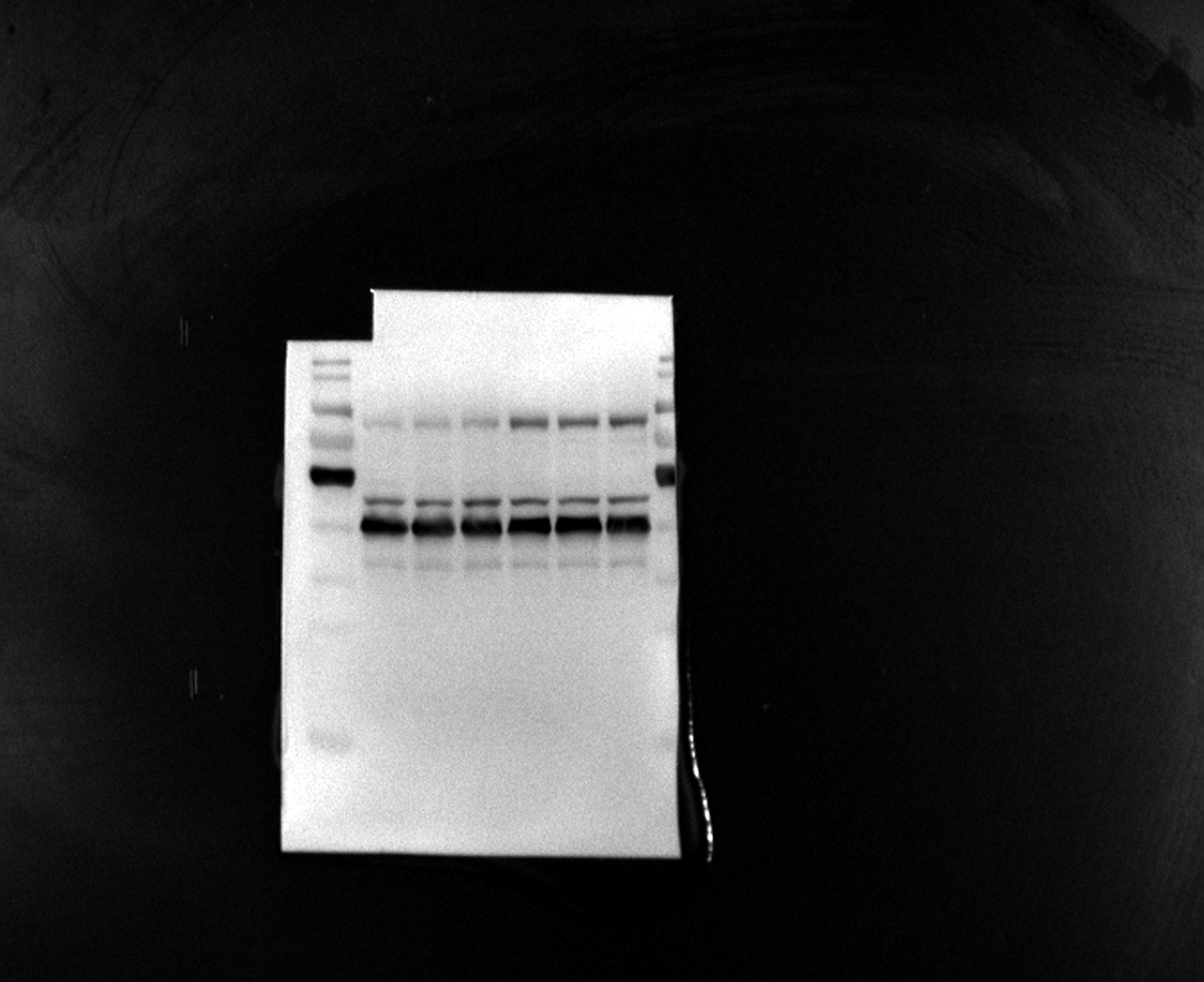

Supplement: Supplementary file 1 [file DataSheet_1.zip › ACT.Tif]

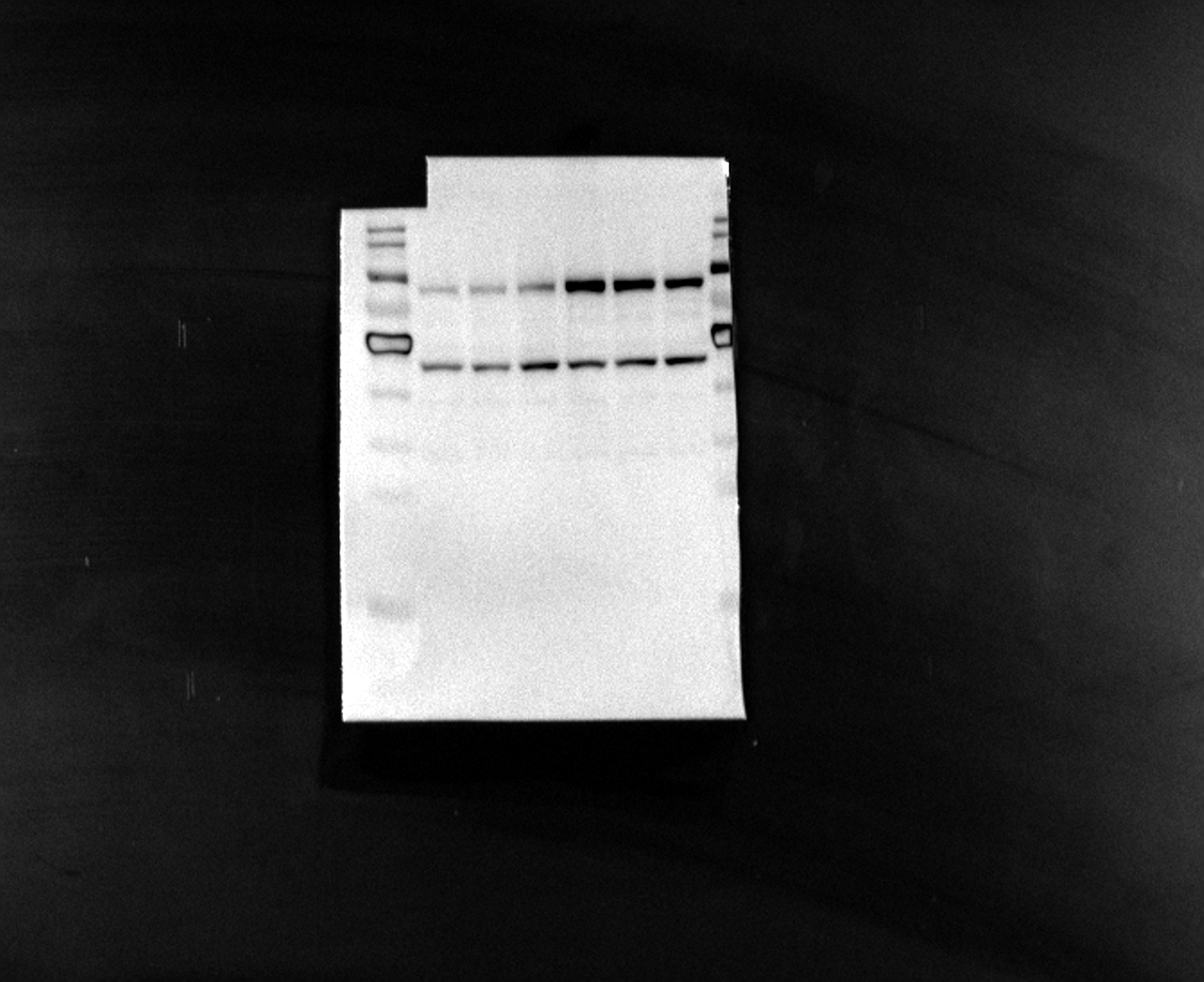

Supplement: Supplementary file 1 [file DataSheet_1.zip › CD73-oe.Tif]

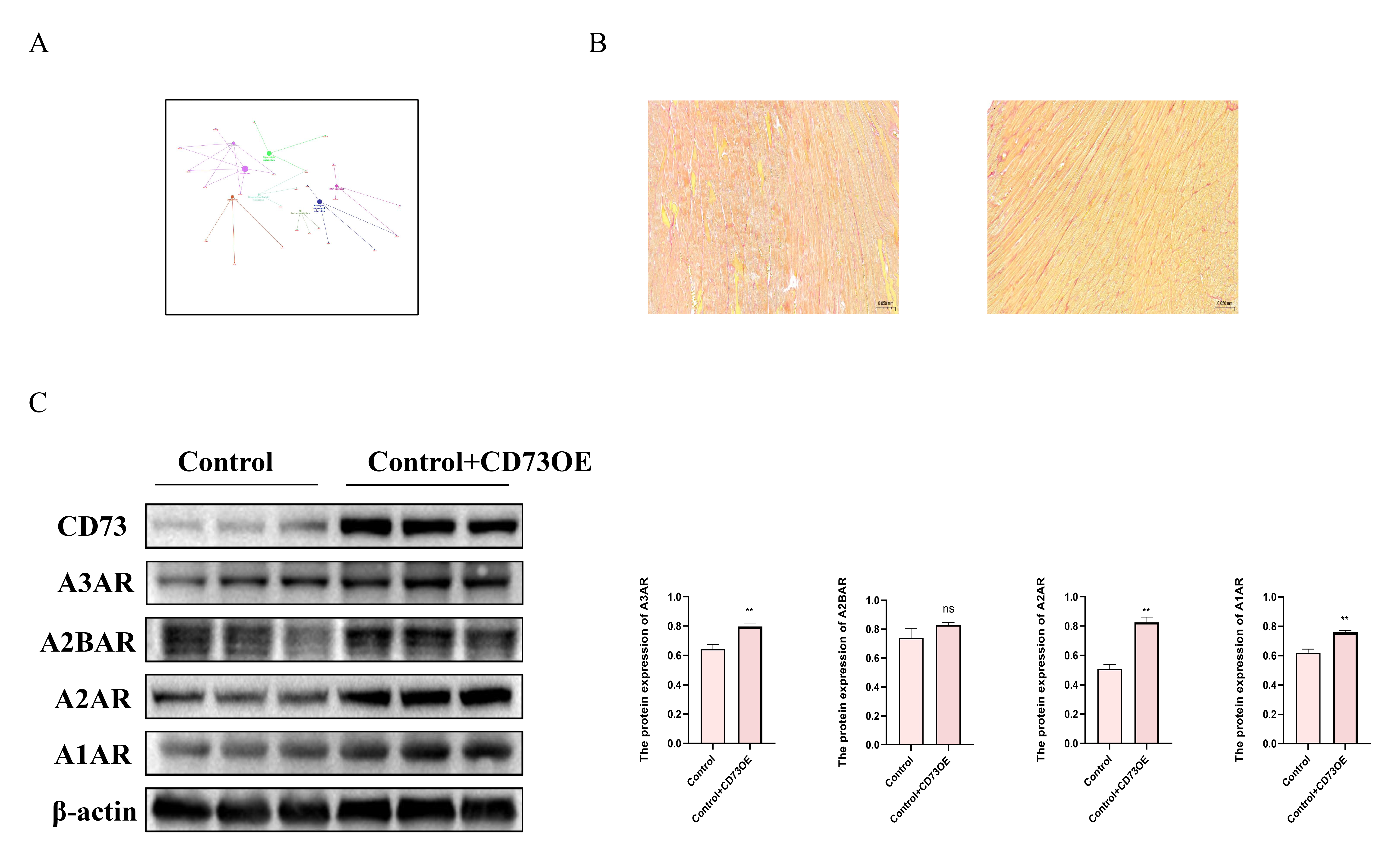

Supplement: Supplementary Figure 1 — Enrichment Analysis of Cardiac Gene Expression and Sirius Red Staining Results of Cardiac Tissue after two weeks of biliary ligation (A) Enrichment Analysis of Cardiac Gene Expression. (B) Representative images of Sirius red staining (Magnification: 400x). (C) Protein expression of CD73, A1AR, A2AR, A2BAR and A3AR in heart tissue(n=6/group). **p < 0.01 vs Sham. [file Image_1.tif]
